# Optic nerve head neurovascular assessments in patients with schizophrenia: A cross‐sectional study

**DOI:** 10.1002/hsr2.2100

**Published:** 2024-05-08

**Authors:** Ramin Daneshvar, Maryam Naghib, Mohammad Reza Fayyazi Bordbar, Farhad Faridhosseini, Marziyeh Fotouhi, Mehrdad Motamed Shariati

**Affiliations:** ^1^ Eye Research Center Mashhad University of Medical Sciences Mashhad Iran; ^2^ Psychiatry and Behavioral Sciences Research Center Mashhad University of Medical Sciences Mashhad Iran

**Keywords:** illness severity, optical coherence tomography angiography, optical imaging, optic disc, schizophrenia

## Abstract

**Objective:**

The retina is a protrusion of the brain, so researchers have recently proposed retinal changes as a new marker for studying central nervous system diseases. To investigate optic nerve head neurovascular structure assessed by optical coherence tomography angiography (OCTA) in schizophrenia compared to healthy subjects.

**Methods:**

The study was conducted from 2019 to 2021 at the Ibn Sina Psychiatric Hospital in Mashhad, Iran. We enrolled 22 hospitalized known cases of schizophrenia, treated with risperidone as an antipsychotic drug, and 22 healthy subjects. The two groups were matched in age and gender. In the schizophrenic group, the positive and negative syndrome scale test was used to assess the illness severity. All subjects underwent complete ophthalmic evaluations and OCTA imaging.

**Results:**

We found that the cup/disc area ratio, vertical cup/disc ratio, and horizontal cup/disc ratio are significantly higher in patients with schizophrenia than in healthy subjects (with *p*‐values of 0.019, 0.015, and 0.022, respectively). No statistically significant difference in the peripapillary retinal nerve fiber layer and vascular parameters of the optic nerve head was observed between schizophrenia and healthy groups.

**Conclusion:**

We found evidence regarding the difference in the optic nerve head tomographic properties in schizophrenia compared to healthy subjects. However, ONH vascular parameters showed no significant difference. More studies are needed for a definite conclusion.

## 1 INTRODUCTION

Schizophrenia is a chronic psychiatric illness associated with changes in sensory perception and cognitive function.^1^ The Epidemiologic Catchment Area study expressed a schizophrenia lifetime prevalence of 0.6%−1.9%.^2^ A significant portion of the affected cases suffer from varying degrees of interpersonal relations, self‐care, and work function loss.

Specific signs and symptoms accompany schizophrenia, and it is thus regarded as a syndrome to some extent. One of the main highlights of this condition is psychotic manifestations, including hallucinations and delusions. Recurring episodes of psychosis are common in patients.

Various models exist which try to clarify the etiology of schizophrenia. Biopsychosocial factors and their interaction with susceptible genetic profiles may eventually end in schizophrenia manifestations.^3^ Some models with emphasis on the genetic‐vascular‐inflammatory aspect of the disease have been proposed in 2005. According to the aforementioned hypothesis, the triggered inflammatory responses that occur subsequently to certain environmental insults, such as infection, trauma, or hypoxia, may disturb microvessel‐astrocytes coupling and lead to dysregulation of cerebral blood flow and dysfunction of the blood‐brain barrier.^4^

Neurotransmitter imbalance has been recognized as one of the main mechanisms of the pathologic changes that take place in schizophrenia. Increased dopamine release capacity in mesocortical and mesolimbic pathways,^5^ elevated brain serotonin levels,^6^ selective dissolution of norepinephrine in certain brain regions,^7^ and the hippocampus GABAergic neuron dysfunction^8^ represents a handful of such imbalances.

Currently, clinical findings through psychiatric evaluations and interviews are the main diagnostic tools. Some experts have suggested specific laboratory tests to help with the diagnosis.^9^ Central nervous system (CNS) imaging modalities have helped rule out alternative diagnoses for more complicated cases and elaborated the anatomical and functional properties of the disease itself. In many structural studies of schizophrenia, some anatomical changes including white matter fiber abnormalities,^10^ ventricles enlargement, and destruction of frontotemporal, thalamocortical, and subcortical‐limbic circuits have been described.^11^

It is no surprise that some experts consider the retina a great candidate for studying neurodegenerative diseases. Retina shares close embryologic, anatomical, and histologic ties to the brain since it is indeed an extension of the CNS. In addition, retinal vascular supply may be affected by the conditions that impress CNS vasculature.^12^ The retinal layers and the retinal vessels can be studied very easily using optical coherence tomography angiography (OCTA) due to media transparency.

**Figure 1:**
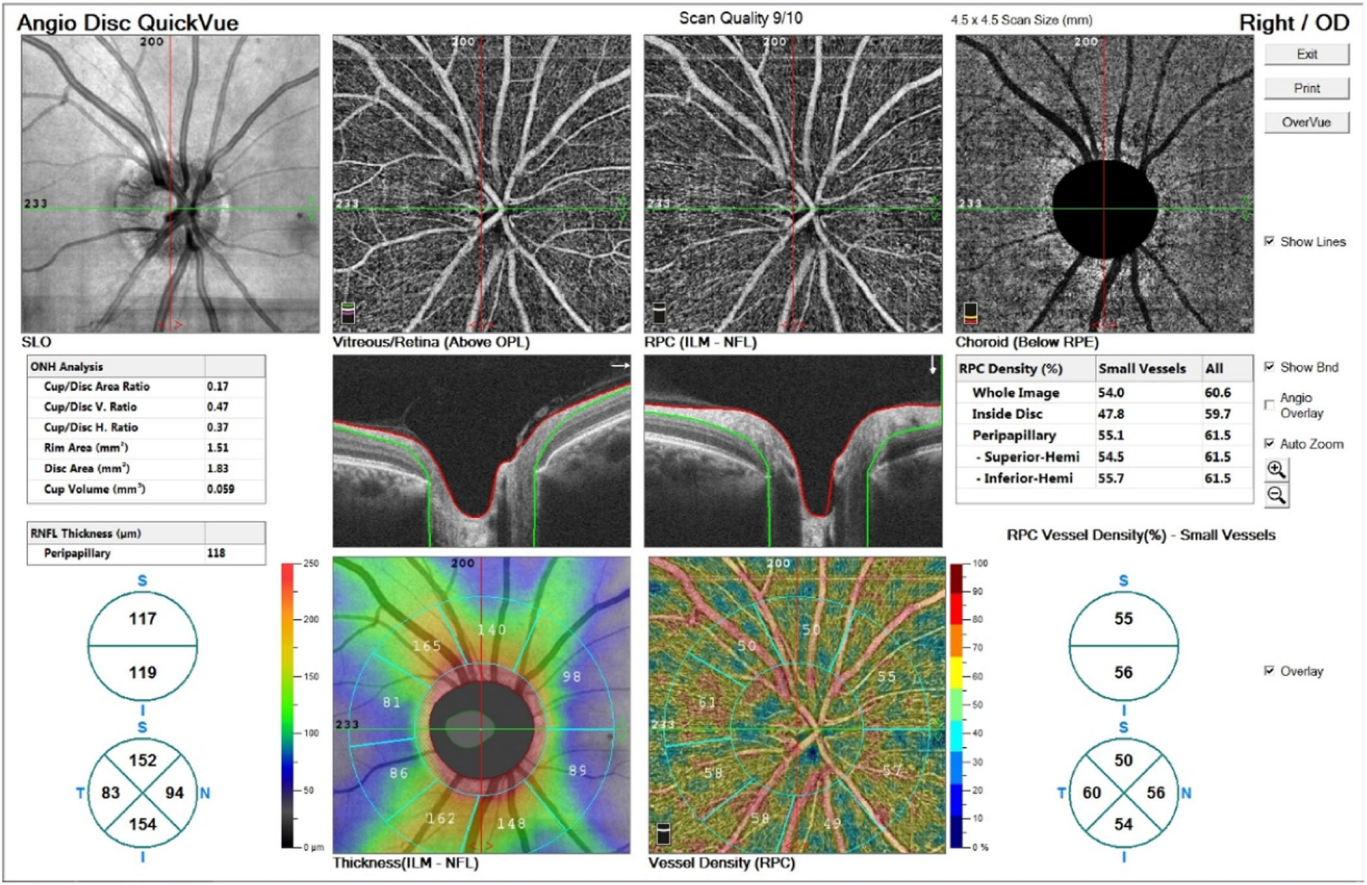
Retinal peripapillary capillary network (RPC) status, optic nerve head tomography (disc size, cup size, and volume, cup‐to‐disc ratio), and thickness of the peripapillary retinal nerve fiber layer (P‐RNFL) in a square of dimensions 4.5 × 4.5 mm to the center of the disc.

OCTA is one of the novel techniques in ophthalmic imaging. This noninvasive modality provides detailed images of retinal, choroidal, and optic nerve head (ONH) blood flow through laser reflection on retinal vessels' red blood cells (Figure 1). Studies on OCTA implications in CNS diseases such as Alzheimer's disease are available, which have revealed controversies. While some studies showed no difference in the macular vascular parameters in Alzheimer's, a greater retinal vessel density has been found in the preclinical stages of Alzheimer's disease in some other studies. However, it seems that retinal vessel densities decreased in chronic disease.^13^, ^14^, ^15^ Besides, some previous studies investigated the choroidal thickness in neurodegenerative diseases such as Parkinson's and multiple systems atrophy (MSA). They showed a significant postural change in choroidal thickness in MSA.^16^

This study investigates OCTA findings of optic nerve head neurovascular structure in schizophrenia compared to healthy subjects.

## 2 METHODS

### 2.1 Participants

We conducted this cross‐sectional study from 2019 to 2021 at the Ibn Sina Psychiatric Hospital in Mashhad, Iran. Twenty‐two hospitalized known cases of schizophrenia, treated with risperidone as an antipsychotic, were enrolled. Two psychiatrists evaluated all of these cases based on DSM‐5 criteria to confirm the diagnosis. We considered consuming certain medications (4‐aminoquinolines, amiodarone, calcium channel blockers, alcohol consumption) or certain medical conditions (uncontrolled hypertension, increased intracranial pressure, seizures, glaucoma), which may affect optic nerve head neurovasculature, as exclusion criteria. We divided the patients with schizophrenia into two subgroups: duration of illness 2 years or less (DITYL) and duration of illness more than 2 years (DIMTY). Data on patients' medical histories, medications, and psychiatric status were recorded beside their demographic data (age, gender).

In this study, we used the positive and negative syndrome scale (PANSS) test to evaluate the disease severity. PANSS was developed in 1986 to assess the severity of symptoms in schizophrenia patients. This test evaluates three distinct domains of schizophrenia symptoms using 30 items (positive and negative symptoms, and general psychopathology, including 7, 7, and 16 items, respectively). Each item is scored up to 7.^17^ We divided schizophrenia patients into four groups based on PANSS total score: mildly ill (up to PANSS score of 58), moderately ill (PANSS score of 59−75), markedly ill (PANSS score of 76−95), and severely ill (PANSS score of 96−116)^18^

### 2.2 Ophthalmic examination

All subjects underwent the following ophthalmic evaluations at the Khatam Anbia Eye Hospital: best‐corrected visual acuity (BCVA) measurement with thumbing E chart, slit‐lamp biomicroscopy, Goldmann applanation tonometry (GAT), complete fundus examination (using a+90D condensing lens), and optic nerve head OCTA (AngioVue RTVue XR Avanti; Optovue; software version 2018.0.0.18) with 4.5 × 4.5 mm scan size. OCTA imaging was performed before dilated fundus examination, as the mydriatics could alter the ONH blood flow.^19^ Any images with a quality index below 6/10 were discarded, and the imaging was repeated. The study excluded participants with a history of pregnancy, drug abuse, systemic illness, ocular disease such as glaucoma, and prior intraocular surgery and ocular trauma. In cases of any significant abnormalities in ophthalmic evaluations (visually significant cataract, epiretinal membrane, etc.), or two imaging with below 6/10 quality indices, the derived data from that eye were excluded from the analysis. Twenty‐two healthy individuals with no previous histories of any psychiatric, systemic, or ophthalmic disorders were enrolled as the control group. None of these subjects had any history of consuming any drugs with potential effects on the optic nerve, vessels, or retinal tissues. This control group was matched in regard to age and sex with the case group. The data from randomly selected one eye of each case was entered in the final analysis.

### 2.3 Statistical analysis

We used Statistical Package for Social Sciences (SPSS) software version 22 (IBM SPSS Statistics; IBM Corporation) for statistical analysis. We used the Shapiro−Wilk test to confirm normal data distribution. Independent samples *t*‐test and *χ* ^2^ test were used to verify the two groups were matched. The statistical significance of the difference in the retinal peripapillary small vessels density, retinal peripapillary all vessels density, small vessels density inside the disc, all vessels density inside the disc, whole image small vessels density, whole image all vessels density, retinal nerve fiber layer (RNFL) thickness and optic nerve head analysis data including rim area, cup volume, horizontal and vertical cup/disc ratio, and cup/disc area ratio between patients with schizophrenia and controls were determined by independent samples *t*‐test or its nonparametric equivalent. We used Pearson's correlation or its nonparametric equivalent, Spearman's correlation, to investigate the relationship between PANSS and OCTA parameters, and Independent‐samples *t*‐test or its nonparametric equivalent to compare OCTA parameters in subgroups of schizophrenia patients based on PANSS scores. The statistical power was set at 80% (type 2 error was set at 0.2). Also, we considered a *p*‐value < 0.05 as the level of statistical significance.

## 3 RESULTS

### 3.1 Descriptive statistics

The study investigates 22 patients with schizophrenia (11 males, 11 females) and 22 healthy subjects (12 males, 10 females) (*p* = 0.763). The mean ± SD age of schizophrenic and healthy participants was 35.86 ± 9.29 and 34.36 ± 6.56 years, respectively (*p* = 0.540). We gathered PANSS score data from 22 participants (schizophrenia group). PANSS score was distributed normally and ranged between 53 and 116 with a mean ± SD of 83.29 ± 13.77 (Table 1). The number of cases in each of the four schizophrenia subgroups, that is, mildly ill, moderately ill, markedly ill, and severely ill, were two, six, 12, and two, respectively. Among the patients with schizophrenia, eight cases were identified as DITYL, and 14 patients had the disease with DIMTY chronology. All of the schizophrenia cases consumed risperidone for treatment with the mean ± SD dose of 4.40 ± 1.40 mg. Some patients occasionally used benzodiazepines, anticholinergics, and other antipsychotics to reduce agitation. While cigarette smoking was recorded in three patients with schizophrenia, none of the control group subjects were cigarette smokers (*p* = 0.73). The participants had no history of consuming certain medications (4‐aminoquinolines, amiodarone, calcium channel blockers, alcohol consumption) or certain medical conditions (uncontrolled hypertension, increased intracranial pressure, seizures, glaucoma), which may affect optic nerve head neurovasculature (Table 2).

**Table 1:** PANSS score of patients with schizophrenia.

PANSS scale	Mean (SD)
Positive	19.48 (4.78)
Negative	20.48 (5.36)
General psychopathology	43.48 (6.67)
Anergia	10.43 (3.30)
Thought disorder	10.90 (2.74)
Activation	5.38 (1.96)
Paranoid/belligerence	9.95 (2.11)
Depression	10.05 (2.16)
PANSS total score	83.29 (13.77)

Abbreviation: PANSS, positive and negative syndrome scale.

**Table 2:** Demographic information.

	Schizophrenia	Control	*p* Value
Sex			
Male	11	12	0.763^a^
Female	11	10	
Age			
Mean (SD)	35.86 ± 9.29	34.36 ± 6.56	0.540^b^
Drug history	Risperidone	No routine medications	–
Visual acuity			
Median	20/20	20/20	–
Intra‐ocular pressure			
Mean (SD)	11.18 (1.59)	10.77 (1.41)	0.498
Previous ophthalmic surgery	No ophthalmic surgery	No ophthalmic surgery	–

^a^ *χ* ^2^ test

^b^ Independent‐samples *t*‐test.

### 3.2 Ocular findings

All of the subjects in both groups had a BCVA of 10/10, and there were no significant ocular conditions with potential impacts on retinal neurovasculature. The range of refractive errors was −2.5 diopter to +1.25 diopter. The mean ± SD for intraocular pressure (IOP) was 10.77 ± 1.41 and 11.18 ± 1.59 mmHg for the control and schizophrenia groups, respectively (*p* = 0.498). Retinal peripapillary small vessels density, retinal peripapillary all vessels density, small vessels density inside the disc, all vessels density inside the disc, whole image small vessels density, whole image all vessels density, RNFL thickness, and optic nerve head analysis data including rim area, disc area, cup volume, horizontal and vertical cup/disc ratio, and cup/disc area ratio were evaluated and compared between the groups. Statistically significant differences exist in horizontal and vertical cup/disc ratio and cup/disc area ratio (*p* = 0.022, *p* = 0.015, *p* = 0.019, respectively) between patients with schizophrenia and healthy subjects (Table 3). We found no significant differences in OCTA parameters between the two genders in control subjects and schizophrenia cases.

**Table 3:** Comparison of optic nerve head and RNFL thickness between patients with schizophrenia and controls.

	Group	Mean	SD	*p* Value
Whole image small vessels density (%)	Schizophrenia	49.44	2.12	0.651
	Control	49.81	3.13	
Whole image of all vessel density (%)	Schizophrenia	55.55	2.14	0.50
	Control	56.05	2.61	
Small vessel density inside the disc (%)	Schizophrenia	46.70	5.31	0.34
	Control	48.28	5.34	
All vessel density inside the disc (%)	Schizophrenia	56.48	4.77	0.33
	Control	57.89	4.62	
Retinal peripapillary small vessel density (%)	Schizophrenia	52.60	2.52	0.58
	Control	52.14	2.89	
Retinal peripapillary all vessel density (%)	Schizophrenia	58.47	2.22	0.89
	Control	58.57	2.54	
Cup/disc area ratio	Schizophrenia	0.22	0.10	0.019*
	Control	0.14	0.99	
Vertical cup/disc ratio	Schizophrenia	0.49	0.11	0.015*
	Control	0.36	0.19	
Horizontal cup/disc ratio	Schizophrenia	0.44	0.11	0.022*
	Control	0.32	0.17	
Rim area (mm²)	Schizophrenia	1.51	0.19	0.55
	Control	1.66	0.27	
Disc area (mm²)	Schizophrenia	1.96	0.29	0.86
	Control	1.95	0.29	
Cup volume (mm³)	Schizophrenia	0.08	0.07	0.104
	Control	0.05	0.05	
Peripapillary RNFL thickness (μm)	Schizophrenia	108.00	9.89	0.063
	Control	113.77	10.65	

Abbreviation: RNFL, retinal nerve fiber layer.

* Considered as statistically significant.

We observed no statistically significant difference in ONH analysis and OCTA parameters between DITYL and DIMTY subgroups (Table 4).

**Table 4:** Comparison of optic nerve head and RNFL between DITYL and DIMTY.

	Subgroup	*N*	Mean	SD	*p* Value
Cup/disc area ratio	DITYL	8	0.26	0.11	0.493
	DIMTY	14	0.21	0.10	
Vertical cup/disc ratio	DITYL	8	0.52	0.11	0.627
	DIMTY	14	0.49	0.11	
Horizontal cup/disc ratio	DITYL	8	0.47	0.12	0.443
	DIMTY	14	0.42	0.11	
Rim area (mm²)	DITYL	8	1.52	0.17	0.804
	DIMTY	14	1.49	0.23	
Disc area (mm²)	DITYL	8	2.08	0.33	0.333
	DIMTY	14	1.91	0.28	
Cup volume (mm³)	DITYL	8	0.11	0.08	0.423
	DIMTY	14	0.07	0.07	
Peripapillary RNFL thickness (μm)	DITYL	8	109.17	9.56	0.512
	DIMTY	14	106.00	7.71	

Abbreviation: RNFL, retinal nerve fiber layer.

We did not include mildly and severely ill patients in statistical analyses due to the small number of cases in each subgroup. As we showed in Table 5, retinal peripapillary small vessel density and retinal peripapillary all vessel density were significantly higher in markedly ill patients than in moderately ill cases (*p* = 0.009, *p* = 0.014).

**Table 5:** Comparison of optic nerve head vessels between moderately ill and markedly ill subgroup.

	Subgroup	*N*	Mean	SD	*p* Value
Whole image small vessels density (%)	Moderately ill	6	48.30	1.87	0.070
	Markedly ill	12	50.37	1.93	
Whole image of all vessel density (%)	Moderately ill	6	54.40	1.62	0.065
	Markedly ill	12	56.54	2.07	
Small vessel density inside the disc (%)	Moderately ill	6	45.88	4.94	0.539
	Markedly ill	12	47.89	6.17	
All vessel density inside the disc (%)	Moderately ill	6	55.92	3.53	0.582
	Markedly ill	12	57.53	5.80	
Retinal peripapillary small vessel density (%)	Moderately ill	6	50.94	2.03	0.009*
	Markedly ill	12	53.89	1.64	
Retinal peripapillary all vessel density (%)	Moderately ill	6	57.08	1.62	0.014*
	Markedly ill	12	59.76	1.75	

* Considered as statistically significant.

We also evaluated the probable correlation between PANSS parameters and OCTA data. Considering the normal distribution of data, Pearson's correlation was utilized. There was a significant positive correlation between small vessel density inside the disc and PANSS depression (*r* = 0.49, *p* = 0.04). We could not find any significant correlations between the other parameters.

## 4 DISCUSSION

We aimed to investigate neural and vascular structures of optic nerve heads in patients with schizophrenia. We used OCT‐A as a noninvasive imaging modality for this purpose. Other studies have used the eye as a model to assess neurovascular changes in some psychiatric illnesses. In a study, an evaluation of retinal changes in bipolar disorder using OCT showed a peripapillary reduction of the RNFL. They concluded evaluation of RNFL with OCT might be a useful tool to monitor disease progression.^20^ Edyta et al. in a study showed that retinal microvasculature assessment can be potentially useful in patients with bipolar disorder and schizophrenia.^21^ Furthermore, choroidal thickness alterations in neurodegenerative and psychotic disorders have been investigated previously.^16^, ^22^ Changes in choroidal microvasculature have been shown in patients with first‐episode psychosis.^22^ So far, no study has been performed on the evaluation of neurovascular structures in schizophrenia patients in Iran.

In this study, 22 patients with schizophrenia and 22 healthy control subjects participated, and the two groups were matched in age and gender. We used the PANSS test to evaluate the disease severity. Optic nerve head properties, including cup/disc area ratio, and vertical and horizontal cup/disc, were measured. Also, we measured vessel densities in the peripapillary region, inside the optic disc, and in the whole image of the optic nerve head area. There has been some evidence that vascular inflammatory processes play some roles in schizophrenia pathogenesis, and this forms the basis of our attention to optic nerve vascular densities.^23^ We demonstrated that the cup/disc area ratio, and vertical and horizontal cup/disc ratio are significantly higher in patients with schizophrenia than healthy individuals. No statistically significant difference in peripapillary RNFL thickness and vascular parameters in the optic nerve head was observed between the two groups.

In 2017, Silverstein et al. studied optic nerves in 32 patients with schizophrenia and 32 healthy control subjects. Their results showed the cup/disc ratio was significantly higher in the schizophrenia group than in the controls. RNFL thickness did not differ significantly between the two groups.^24^ These results are compatible with our study findings.

In 2012, Cabezon et al. investigated the retina properties in 30 schizophrenia cases and 30 healthy subjects. They showed significant increases in cup/disc area ratio, and vertical and horizontal cup/disc ratio in patients with schizophrenia. A decrease in RNFL thickness in patients with schizophrenia was recorded, for which the authors proposed the possibility of the neurodegenerative process in action.^25^

While decreased peripapillary RNFL thickness in patients with schizophrenia has been reported in some studies,^25^, ^26^, ^27^ other studies did not end with similar results.^24^, ^28^ Interestingly, several authors have proposed that antipsychotic medications and the disease duration may affect RNFL thickness.^27^, ^29^

Based on our study, there are no statistically significant differences in small vessel densities and whole peripapillary vascular densities between schizophrenic and healthy subjects. It must be noted that these parameters are higher in markedly ill patients compared to moderately ill cases. However, these distribution patterns might be due to the small number of cases in schizophrenia patient subgroups.

Previous results on peripapillary vascular densities in patients with schizophrenia have been controversial. Using OCTA, Akda et al. in 2019 compared retinal microvasculature in patients with schizophrenia and healthy individuals. Their findings showed a statistically significant decrease in optic nerve capillary density in patients with schizophrenia.^27^ In 2020, Asanad et al. compared the retinal thickness and its vascular properties between 39 patients with schizophrenia and 27 healthy subjects using OCT and OCTA. They reported a significant decrease in temporal quadrant peripapillary perfusion density in schizophrenia patients,^26^ which is not consistent with our results.

Increased cup‐to‐disc ratio can be related to different underlying processes including RNFL degeneration and thinning, vascular disorders such as retinal/optic nerve capillary attenuation, or increased optic nerve head diameter.^30^ Various causes, such as drug toxicity, increased IOP, ischemic vascular processes, and neurodegenerative disorders, have been suggested for RNFL thinning. One may wonder if the difference in cup ratios observed is due to the optic nerve atrophy secondary to unmentioned head trauma or similar instances (e.g., following convulsions/ECT, etc.) in the schizophrenia group. The lack of a significant difference in RNFL does make that assumption less likely.

Our results suggest that the destruction of peripapillary neural fibers cannot be regarded as the cause of the increased cup‐to‐disc ratio in patients with schizophrenia. According to Ascaso et al., retinal inflammation in acute psychotic episodes may mask retina neural destruction in patients^31^; we did not encounter any clinical evidence of inflammation in the optic head nerve or retina in any of the cases. More studies are needed to unveil the possible role of inflammation in schizophrenia pathogenesis. This study showed no significant differences in terms of optic nerve head size and IOP between patients with schizophrenia and healthy subjects. The gold standard for measuring IOP is GAT. However, a number of variables, including central corneal thickness, curvature (Km), and structure, might affect its accuracy.^32^ On the other hand, vascular densities did not show any differences between the healthy subjects and schizophrenia cases, which does not support optic nerve ischemia in the schizophrenic cases. Increased cup‐to‐disc ratio has been reported in other CNS disorders such as Alzheimer's disease and multiple sclerosis. This fact may suggest similar pathophysiologic aspects of these neuropsychiatric disorders, independent of peripapillary RNFL thinning.^33^

Numerous studies have shown that the RNFL thickness is significantly lower in patients with schizophrenia than in normal subjects. This finding may be due to the presence of some other factors, such as the presence of hypertension or diabetes, smoking, age‐related changes, or ethnicity.^34^

Considering the current study, certain limitations come to mind. Cross‐sectional studies have their limitations for statistical inference. Patients' drug histories before their hospital admission, were not recorded in our database. Our study suffers from a relatively small sample size, especially regarding the schizophrenia subgroups, which affects the study's statistical power Also, the detailed past medical history of the patients has not been recorded.

We suggest that extensive studies with longitudinal and cohort designs be carried out on this topic. Larger sample sizes may help clarify some of the unanswered questions in this field. Furthermore, higher cup‐to‐disc ratios may be a morphological finding in people with schizophrenia, and examining family members of these patients may be helpful.

The results of this study showed a significantly higher cup/disc area ratio, vertical cup/disc ratio, and horizontal cup/disc ratio in patients with schizophrenia compared to healthy subjects. However, vascular parameters of the ONH showed no significant difference between the two groups. Differences in papillary excavation are not clinically relevant, and we found no differences in the RNFL thickness or papillary excavation volume. Regarding the small sample size, the differences between both study groups may be due to physiological papillary excavation size changes in the general population.

## AUTHOR CONTRIBUTIONS

**Ramin Daneshvar**: Conceptualization; writing—review and editing; supervision. **Maryam Naghib**: Investigation; writing—original draft; writing—review and editing; data curation. **Mohammad Reza Fayyazi Bordbar**: Supervision; project administration; writing—review and editing. **Farhad Faridhosseini**: supervision; project administration; writing—review and editing. **Marziyeh Fotouhi**: Data curation; investigation. **Mehrdad Motamed Shariati**: Conceptualization; investigation; writing—original draft; writing—review and editing; formal analysis.

## CONFLICT OF INTEREST STATEMENT

The authors declare no conflict of interest.

## ETHICS STATEMENT

This study is done according to the declaration of Helsinki and it was approved by the institutional review board and ethics committee of the Mashhad University of Medical Sciences (approval number: IR.MUMS.MEDICAL.REC.1399.696). Consent to participate in the study was acquired from the patients or their legal guardians.

## TRANSPARENCY STATEMENT

The lead author, Mehrdad Motamed Shariati, affirms that this manuscript is an honest, accurate, and transparent account of the study being reported, that no important aspects of the study have been omitted, and that any discrepancies from the study as planned (and, if relevant, registered) have been explained.

## Data Availability

The data that support the findings of this study are available on request from the corresponding author. The data are not publicly available due to privacy or ethical restrictions.
